# The relationship between upper airway collapse and the severity of obstructive sleep apnea syndrome: a chart review

**DOI:** 10.1186/s40463-015-0086-2

**Published:** 2015-09-04

**Authors:** Russell N. Schwartz, Richard J. Payne, Véronique-Isabelle Forest, Michael P. Hier, Amanda Fanous, Camille Vallée-Gravel

**Affiliations:** Faculty of Science, McGill University, 845 Rue Sherbrooke West, Montréal, QC Canada; Department of Otolaryngology-Head and Neck Surgery, Sir Mortimer B. Davis-Jewish General Hospital, McGill University, 3755 Côte Ste-Catherine Road, Montreal, QC Canada; Department of Otorhinolaryngology Adult (Otl) (Ent) (Surgery), Royal Victoria Hospital, McGill University Health Center, 687 Pine Avenue West, Montreal, QC Canada; Department of Otolaryngology-Head and Neck Surgery, McGill Executive Institute, McGill University, 1001 Rue Sherbrooke West, Montreal, QC Canada; Faculty of Medicine, McGill University, 845 Rue Sherbrooke West, Montréal, QC Canada

**Keywords:** Obstructive sleep apnea syndrome, Mueller maneuver, Upper airway, Sleep study, Apnea-hypopnea index

## Abstract

**Background:**

We sought to determine the ability of the endoscopic Mueller maneuver (MM) to predict the severity of OSAS based on upper airway (UA) collapse.

**Methods:**

This chart review retrospectively analyzed the results of endoscopic Mueller maneuvers examining the UA on 506 patients suspected of having OSAS. There were 3 areas of UA collapse that were evaluated: velopharynx (VP), base of tongue (BOT), and lateral pharyngeal walls (LPW). A sleep study was done after the examination to assess the severity of OSAS based on the apnea-hypopnea index (AHI).

**Results:**

A total of 506 patients met criteria for OSAS, with 194 mild cases (5 ≤ AHI < 15), 163 moderate cases (15 ≤ AHI < 30) and 149 severe cases (30 ≤ AHI). At the VP, 30 patients had minimal collapse (mean AHI = 17); 41 patients had moderate VP collapse (mean AHI = 25); 392 patients had severe VP collapse (mean AHI = 27). At the BOT, 144 patients had minimal collapse (mean AHI = 19); 187 patients had moderate BOT collapse (mean AHI = 24); 175 patients had severe BOT collapse (mean AHI = 33). At the LPW, 158 patients had minimal collapse (mean AHI = 20); 109 patients had moderate LPW collapse (mean AHI = 25); 120 patients had severe LPW collapse (mean AHI =33). The correlations found between VP collapse, BOT collapse, and LPW collapse and OSAS severity were: *r* = 0.069 (95 % CI; −0.022, 0.16), *r* = 0.26 (95 % CI; 0.18, 0.34) and *r* = 0.22 (95 % CI; 0.12, 0.31), respectively.

**Conclusions:**

In this study, the degree of collapse of the UA at all levels, especially at the BOT and LPW levels, correlate significantly with the severity of OSAS. The Mueller maneuver helped identify patients with severe sleep apnea based on UA collapse. The MM cannot be used to diagnose OSAS, but can be a valuable tool to help the physician estimate the severity of sleep apnea and the urgency to obtain a sleep study.

## Introduction

Obstructive Sleep Apnea Syndrome (OSAS) is a condition affecting 14 % of males and 5 % of females in adults 30–70 years of age [1]. OSAS is characterized by recurrent partial or complete obstruction of the upper airway (UA) during sleep, leading to cessation of airflow, intermittent and recurrent hypoxemia and sleep fragmentation. The sites of obstruction are generally divided in three anatomical areas, which are the velopharynx (VP), the base of the tongue (BOT) and the lateral pharyngeal walls (LPW).

A sleep study is required to diagnose obstructive sleep apnea syndrome and determine its severity. Unfortunately, sleep studies are labor-intensive and waiting times to get a sleep study in a hospital can be very long, even up to a few years. Therefore, diagnosis and treatment of the condition can be delayed significantly. This can be a real concern, especially if a patient has moderate to severe OSAS and is left untreated while waiting for the test. It is still a challenge for physicians to identify which patients would need a more urgent sleep study based on history or questionnaires alone. This study looked at the ability of the endoscopic Mueller maneuver (MM) to help predict the severity of OSAS based on UA collapse at its different levels.

The MM is a minimally invasive examination tool which has significant value due to its ease of use in clinical settings. The maneuver is done on an awake patient and can be conducted in less than a two-minute time span. The MM has mixed evidence regarding its effectiveness of predicting OSAS. Studies such as Woodson and Haganuma [2], as well as Friedman et al. [3] found no positive correlation between MM performance and OSAS. In contrast to these findings, Dreher et al. [4] found the Mueller maneuver to be predictive of OSAS based on the degree of obstruction at base of tongue and velum. The authors rationalized the divergent findings to be due to possible differences in patient cooperation as a result of the MM performed on awake patients. In the present study, the goal is to determine how well the MM maneuver can predict OSAS by observing how airway obstruction at the levels of the VP, BOT and LPW during the procedure relates to the severity of OSAS, as measured by AHI.

## Methods

This study is a retrospective chart review of 506 consecutive patients referred to a McGill University Affiliated Teaching Site otolaryngology office in Montreal, Canada for snoring, and/or suspected obstructive sleep apnea syndrome. The review (CR14-62) has been approved by the Research Ethics Committee at the Sir Mortimer B. Davis-Jewish General Hospital.

All patients completed an Epworth Sleepiness Scale (ESS) [5] and had a full head and neck examination by one of three experienced physicians, including flexible nasopharyngolaryngoscopy and MM. Patients then underwent a level 3 cardiorespiratory polygraphy that was scored using American Academy of Sleep Medicine (AASM) criteria [6]. These studies were done using a Philips Stardust II Sleep Recorder. The recordings included monitoring of respiratory effort, oro-nasal pressure, oximetry, body position, snoring sound and pulse rate channels. The number of central apneas, obstructive apneas, mixed apneas and hypopneas were measured and added to form the apnea-hypopnea index. For hypopnea, the definition used was a reduction in nasal airflow ≥30 % with an associated desaturation ≥ 3 %. The severity of OSAS was graded following a scale based on the AHI: 5 ≤ AHI < 15 (mild), 15 ≤ AHI < 30 (moderate) and 30 ≤ AHI (severe).

The measures investigated were the relations between the three sites of obstruction based on the Mueller maneuver and BMI, and OSAS severity (AHI). The relation of OSAS severity with ESS was also investigated.

Data analysis was done using SAS, version 9.3 (Cary, NC). Continuous variables were presented as means with a standard deviation (SD). The correlation between the sites of obstruction with BMI, ESS, and AHI was done using a Spearman’s correlation test using a 95 % confidence interval (CI). A multivariate multiple logistic regression analysis was done to determine if the degree of collapse using the MM predicted OSA severity and the effects of BMI, ESS and age on these associations were analyzed.

Patients who underwent a Mueller maneuver and a sleep study were included in the study. No patients were excluded on the basis of gender, age, or BMI. Patients who did not meet criteria for OSAS diagnosis (AHI <5) were excluded from the study. Patients’ charts lacking data from the MM examination of airway obstruction were excluded.

There were no differences in demographic (age, BMI), ESS, or sleep characteristics (AHI) between the patients who lacked data on airway obstruction and those who were included in the study. There was also no bias towards taking measurements only in patients that had some degree of baseline airway narrowing at the UA levels.

### Endoscopic Mueller maneuver

The Mueller maneuver attempts to emulate the collapse of the UA during sleep [7]. A flexible laryngoscope is inserted through the nose of the patient into his UA and the patient is asked to perform a series of reverse Valsalvas. The examination is of short duration (less than 2 min) and is recorded. All recordings of the examinations for each patient was saved and backed up on a hard drive for later use by the scorers. The scorers were able to go back to the recordings after the consultation with the patient to insure the accuracy of their scores on the Mueller maneuver. Assessors estimated a precise percentage score for the degree of obstruction of the VP, BOT, and LPW. The scores were then sorted semi-quantitatively following a scale based on the percentage of obstruction: ≤50 % (minimal), 51–75 % (moderate) and 76–100 % (severe).

### Data analysis

The data from the sleep studies and MMs were collated along with the age, gender, height, weight, and BMI. A Spearman’s correlation coefficient was calculated to evaluate the association between the areas of collapse and the severity of OSAS. If a correlation were to be found, it would then be concluded that the area of obstruction that correlated would be considered as contributing to the severity of OSAS.

## Results

There were 399 male (79 %) and 107 female (21 %) subjects. The mean age was 49 years old (SD = 12) and ages ranged from 16 to 90 years. The mean BMI was 29 kg/m^2^ (SD = 5) and ranged from 19 to 55 kg/m^2^. The mean ESS was 9 (SD = 5) and ranged from 0 to 24.

BMI and VP collapse correlate, *r* = 0.15 (95 % CI; 0.063, 0.24). BMI and BOT collapse correlate, *r* = 0.31 (95 % CI; 0.22, 0.38). BMI and LPW collapse correlate, *r* = 0.25 (95 % CI; 0.15, 0.34). Overall, BMI and AHI correlate, *r* = 0.42 (95 % CI; 0.35, 0.49).

At the VP, females had a mean collapse of 86 % (SD = 15), while males had a mean of 85 % (SD = 16). At the base of tongue, females had a mean collapse of 60 % (SD = 20), while males had a mean of 65 % (SD = 20). At the lateral pharyngeal wall, females had a mean collapse of 55 % (SD = 22), while males had a mean of 60 % (SD = 22). Overall, females had a mean AHI of 20 (SD = 18), while males had a mean AHI of 27 (SD = 20).

### Sleep study screening findings

AHI values ranged from 5 to 122 events per hour with a mean of 26 (SD = 20) events per hour. Of the 506 total patients, 194 (38 %) had mild OSAS, 163 (32 %) had moderate OSAS, and 149 (30 %) had severe OSAS (Table 1).

**Table 1:** Distribution of the severity of OSAS at each UA level

OSAS severity; AHI	n (%)	Mean VP collapse (%; ± SD)	Mean BOT collapse (%; ± SD)	Mean LPW collapse (%; ± SD)
Mild; 5 to 15 events per hour	194(38)	85(±18)	59(±20)	54(±21)
Moderate; 15 to 30 events per hour	163(32)	84(±17)	63(±20)	59(±22)
Severe; 30+ events per hour	149(30)	87(±12)	72(±18)	67(±23)

This table shows the association between each AHI category (mild = 5 ≤ AHI < 15; moderate = 15 ≤ AHI < 30; severe = 30 ≤ AHI). n (%) = sub-sample size (percentage of overall sample). The mean collapse at each UA is shown at each level of AHI

### Endoscopic Mueller maneuver findings

Of the 506 patient charts, 463 had data for VP collapse, 506 had data for BOT collapse, and 387 had data for LPW collapse. Of the 463 patients with data on VP level, 30 had a minimal airway collapse at that level, 41 had a moderate airway collapse and 392 had a severe airway collapse. Among the 506 patients with BOT collapse, 144 had a minimal collapse, 187 had a moderate collapse and 175 had a severe collapse. Of 387 patients with data for obstruction at the LPW, 158 had a minimal collapse, 109 had a moderate collapse and 120 had a severe collapse (Table 2).

**Table 2:** Mean AHI according to the level and degree of collapse

Velopharynx collapse	n (%)	Mean AHI (events per hour)	Standard deviation (events per hour)
Minimal	30 (6)	17	11
Moderate	41 (9)	25	15
Significant	392 (85)	27	21
*r* = 0.069 (95 % CI; −0.022, 0.16)			
Base of Tongue Collapse			
Minimal	144 (28)	19	12
Moderate	187 (37)	24	19
Significant	175 (35)	33	23
*r* = 0.26 (95 % CI; 0.18, 0.34)			
Lateral Pharyngeal Wall Collapse			
Minimal	158 (41)	20	16
Moderate	109 (28)	25	19
Significant	120 (31)	33	25
*r* = 0.22 (95 % CI; 0.12, 0.31)			

This table shows the mean AHI value at each degree of UA collapse (minimal = airway collapse of 0-50 %; moderate = airway collapse of 51–75 %; severe = airway collapse of 76–100 %). r and CI values are marked after each analysis

### Correlation between areas of collapse and OSAS severity

At the VP, patients with minimal collapse had a mean AHI of 17 (SD = 10) events per hour; patients with moderate collapse had a mean AHI of 25 (SD = 15) events per hour; and patients with severe collapse had a mean AHI of 27 (SD = 21) events per hour. This correlation was *r* = 0.069 (95 % CI; −0.022, 0.16) (Table 2).

A positive correlation was observed under a comparative analysis between BOT percentages of collapse and AHI values, *r* = 0.26 (95 % CI; 0.18, 0.34). There was a significant rise in the mean AHI for every collapse degree. Patients with a minimal collapse at the BOT had a mean AHI of 19 (SD = 12) events per hour; patients with moderate collapse had a mean AHI of 24 (SD = 19) events per hour; patients with severe collapse had a mean AHI of 33 (SD = 24) events per hour (Table 2).

At the LPW, there was a correlation between degree of collapse and OSAS severity, *r* = 0.22 (95 % CI; 0.12, 0.31). Patients with minimal collapse had a mean AHI of 20 (SD = 16) events per hour; patients with moderate collapse had a mean AHI of 25 (SD = 19) events per hour; patients with severe collapse had a mean AHI of 33 (SD = 25) events per hour (Table 2).

### Relation between OSAS measures and Epworth sleepiness scale score

The mean ESS score for all 604 patients was 9 (SD = 5). A positive correlation was found between BOT collapse and ESS scores, *r* = 0.18 (95 % CI; 0.096, 0.26), and between LPW collapse and ESS scores, *r* = 0.10 (95 % CI; 0.0022, 0.20). The association between VP collapse and ESS scores was *r* = 0.0091 (95 % CI; −0.082, 0.10). ESS scores and AHI were positively correlated, *r* = 0.19 (95 % CI; 0.11, 0.27).

### Multiple logistic regression analysis of VP, BOT and LPW on AHI outcomes

Patients with severe compared to those with minimal VP collapse have an odds ratio (OR) of 2.75 (95 % CI; 0.99, 7.67) of being in the severe AHI category and OR = 1.64 (95 % CI; 0.68, 3.92) of being in the moderate AHI category. Patients with moderate compared to those with minimal VP collapse have OR = 2.35 (95 % CI; 0.64, 8.73) of being in the severe AHI category and OR = 3.10 (95 % CI; 1.04, 9.30) of being in the moderate AHI category (Table 3).

**Table 3:** VP odds ratio estimates

Effect	AHI	Point estimate	95 % confidence limits	
VP severe vs mild	severe	2.751	0.987	7.669
VP severe vs mild	moderate	1.636	0.682	3.921
VP moderate vs mild	severe	2.354	0.635	8.725
VP moderate vs mild	moderate	3.106	1.037	9.304

This table shows the odds ratio estimates of OSAS severity (based on AHI) for different severities of VP collapse

Patients with severe compared to those with minimal BOT collapse have OR = 5.39 (95 % CI; 2.98, 9.73) of being in the severe AHI category and OR = 1.77 (95 % CI; 1.03, 3.05) of being in the moderate AHI category. Patients with moderate compared to those with minimal BOT collapse have OR = 1.77 (95 % CI; 0.98, 3.19) of being in the severe AHI category and OR = 1.21 (95 % CI; 0.74, 1.99) of being in the moderate AHI category (Table 4).

**Table 4:** BOT odds ratio estimates

Effect	AHI	Point estimate	95 % confidence limits	
BOT severe vs mild	severe	5.386	2.983	9.727
BOT severe vs mild	moderate	1.773	1.030	3.050
BOT moderate vs mild	severe	1.769	0.982	3.186
BOT moderate vs mild	moderate	1.212	0.740	1.985

This table shows the odds ratio estimates of OSAS severity (based on AHI) for different severities of BOT collapse

Patients with severe compared to those with minimal LPW collapse have OR = 3.91 (95 % CI; 2.12, 7.19) of being in the severe AHI category and OR = 1.57 (95 % CI; 0.87, 2.82) of being in the moderate AHI category. Patients with moderate compared to those with minimal LPW collapse have OR = 1.96 (95 % CI; 1.04, 3.68) of being in the severe AHI category and OR = 1.27 (95 % CI; 0.72, 2.24) of being in the moderate AHI category (Table 5).

**Table 5:** LPW odds ratio estimates

Effect	AHI	Point estimate	95 % confidence limits	
LPW severe vs mild	severe	3.905	2.121	7.187
LPW severe vs mild	moderate	1.568	0.873	2.817
LPW moderate vs mild	severe	1.960	1.043	3.682
LPW moderate vs mild	moderate	1.269	0.720	2.238

This table shows the odds ratio estimates of OSAS severity (based on AHI) for different severities of LPW collapse

### Multiple logistic regression analysis of VP, BOT and LPW on AHI outcomes and the effects of BMI, age, and ESS

Accounting for BMI, age and ESS, patients with severe compared to those with minimal VP collapse have OR = 1.77 (95 % CI; 0.60, 5.20) of being in the severe AHI category and OR = 1.44 (95 % CI; 0.59, 3.49) of being in the moderate AHI category. Patients with moderate compared to those with minimal VP collapse have OR = 1.66 (95 % CI; 0.42, 6.63) of being in the severe AHI category and OR = 2.83 (95 % CI; 0.93, 8.57) of being in the moderate AHI category (Table 6).

**Table 6:** VP odds ratio estimates accounting for BMI, age and ESS

Effect	AHI	Point estimate	95 % confidence limits	
VP severe vs mild	severe	1.773	0.604	5.204
VP severe vs mild	moderate	1.439	0.593	3.491
VP moderate vs mild	severe	1.662	0.417	6.631
VP moderate vs mild	moderate	2.827	0.932	8.574
BMI	severe	1.165	1.108	1.224
BMI	moderate	1.052	1.002	1.104
Age	severe	1.024	1.002	1.046
Age	moderate	1.017	0.998	1.037
ESS	severe	1.070	1.022	1.120
ESS	moderate	1.024	0.980	1.070

This table shows the odds ratio estimates of OSAS severity (based on AHI) for different severities of VP collapse after accounting for BMI, age and ESS. It also indicates the odds ratio estimates of OSAS severity (based on AHI) of BMI, age and ESS individually

Accounting for BMI, age and ESS, patients with severe compared to those with minimal BOT collapse have OR = 3.18 (95 % CI; 1.69, 5.99) of being in the severe AHI category and OR = 1.54 (95 % CI; 0.87, 2.72) of being in the moderate AHI category. Patients with moderate compared to those with minimal BOT collapse have OR = 1.40 (95 % CI; 0.75, 2.60) of being in the severe AHI category and OR = 1.15 (95 % CI; 0.70, 1.90) of being in the moderate AHI category (Table 7).

**Table 7:** BOT odds ratio estimates accounting for BMI, age and ESS

Effect	AHI	Point estimate	95 % confidence limits	
BOT severe vs mild	severe	3.177	1.686	5.987
BOT severe vs mild	moderate	1.536	0.869	2.716
BOT moderate vs mild	severe	1.400	0.754	2.596
BOT moderate vs mild	moderate	1.147	0.692	1.899
BMI	severe	1.151	1.095	1.210
BMI	moderate	1.046	0.997	1.097
Age	severe	1.032	1.010	1.053
Age	moderate	1.022	1.003	1.042
ESS	severe	1.055	1.008	1.105
ESS	moderate	1.031	0.988	1.076

This table shows the odds ratio estimates of OSAS severity (based on AHI) for different severities of BOT collapse after accounting for BMI, age and ESS. It also indicates the odds ratio estimates of OSAS severity (based on AHI) of BMI, age and ESS individually

Accounting for BMI, age and ESS, patients with severe compared to those with minimal LPW collapse have OR = 2.53 (95 % CI; 1.32, 4.87) of being in the severe AHI category and OR = 1.40 (95 % CI; 0.77, 2.57) of being in the moderate AHI category. Patients with moderate compared to those with minimal LPW collapse have OR = 1.72 (95 % CI; 0.88, 3.33) of being in the severe AHI category and OR = 1.21 (95 % CI; 0.68, 2.16) of being in the moderate AHI category (Table 8).

**Table 8:** LPW odds ratio estimates accounting for BMI, age and ESS

Effect	AHI	Point estimate	95 % confidence limits	
LPW severe vs mild	severe	2.532	1.318	4.866
LPW severe vs mild	moderate	1.404	0.765	2.573
LPW moderate vs mild	severe	1.715	0.884	3.325
LPW moderate vs mild	moderate	1.214	0.682	2.161
BMI	severe	1.152	1.087	1.220
BMI	moderate	1.034	0.979	1.092
Age	severe	1.027	1.003	1.050
Age	moderate	1.020	0.999	1.041
ESS	severe	1.065	1.010	1.123
ESS	moderate	1.047	0.997	1.100

This table shows the odds ratio estimates of OSAS severity (based on AHI) for different severities of LPW collapse after accounting for BMI, age and ESS. It also indicates the odds ratio estimates of OSAS severity (based on AHI) of BMI, age and ESS individually

## Discussion

The Wisconsin Cohort Study found that the prevalence of OSAS in people aged 30–60 years was 9–24 % for males and 4–9 % for females. It is also widely recognized that BMI is a serious risk factor for OSAS. It was mentioned in the Wisconsin Cohort study that a one standard deviation difference in BMI was associated with a 4-fold increase in OSAS prevalence [8]. In our study, BMI was significantly associated with VP, BOT and LPW collapse as well as with AHI. With a predominance of males, the mean age of 49 years, and patients with high BMIs, our cohort is representative of patients with OSAS [9].

Various studies have been conducted in an attempt to find a correlation between the degree and area of UA collapse and the severity of OSAS using different modalities [7, 10]. These modalities include tri-dimensional magnetic resonance imaging (MRI) [11], cine and sleep-MRI [12], computed tomography (CT) [13], critical pressure measurement (Pcrit) [14], negative expiratory pressure technique (NEP) [15], drug induced sleep endoscopy (DISE) [16] and Mueller maneuver [2–4, 7, 10, 17, 18, 21]. Our study aimed at finding such a relationship using the Mueller maneuver.

In this study, the Mueller maneuver was investigated for its predictive value of the severity of OSAS diagnosis. This maneuver is simple, safe, low cost, and can be part of a routine flexible laryngoscopy done during the head and neck examination. Studies have divergent opinions concerning MM statistical reliability. A 1994 study by Petri et al. found no predictive value in the Mueller maneuver for uvulopalatopharyngoplasty (UPPP) success rate [17]. Patients who primarily had retropalatal obstruction as judged by MM had only a 40 % response to UPPP. On the other hand, Li et al. later found clinical value for MM in improving outcomes from UPPP [18]. This study is not looking at the correlation between the MM and surgical outcomes. We were evaluating if the degree of obstruction seen with the MM could correlate with the severity of OSAS to guide the physician and the patients on the urgency to test for OSAS. We believe the MM is an effective procedure to observe upper airway collapse and its degree and we showed that the greater the collapse in the UA, the greater the chance of having more severe OSAS.

It must be noted that because our study involves the subjective evaluations of three different physicians on the degree of upper airway obstruction, there may be inherent inter-observer variation. A recent study by Ramji et al. [19] tested inter- and intra-rater agreement on sixty-one recorded videos of children undergoing a sleep nasopharyngoscopy. The authors concluded that there was validation for this procedure with good inter- and intra-rater agreement. The study was done using non-expert raters who did not perform the sleep nasopharyngoscopy routinely and were at various stages in their otolaryngology career. In the present study, the three physicians who scored the degree of collapse during the Mueller maneuver would be considered expert raters. All three are trained to perform the endoscopy and have routinely performed the procedure over many years. This cannot ensure that inter-rater variation is reliable in the present study; however it gives more confidence that the results were not biased in this manner.

In our study, a scale similar to the one used to grade the degree of obstruction of the UAs in studies on drug-induced sleep endoscopy was used (≤50 % obstruction = minimal collapse, 51–75 % obstruction = moderate collapse, 76–100 % obstruction = significant collapse). The degree of UA obstruction correlated with AHI at each level studied, mainly at the BOT and LPW. The VP level discriminates weakly compared to the other two levels and this may be explained by the fact that the velum collapses in the majority of patients with OSAS.

From the multivariate multiple logistic regression analysis, it is clear that the strongest effects are seen at the BOT and LPW levels. More specifically, there is a higher likelihood that a patient with severe BOT collapse compared to one with minimal BOT collapse would have severe OSAS. The analysis shows that, while other variables such as BMI, age and ESS scores accentuate this effect, the conclusions still hold true above and beyond these variables. In addition, there is a higher likelihood that a patient with severe LPW collapse compared to one with minimal LPW collapse would have severe OSAS. The analysis also shows that this holds true when accounting for variables such as BMI, age, and ESS scores.

The regression analysis results show significant odds ratios for predicting severe OSAS based on BOT and LPW observations of collapse. These results are important to the purpose of the study, as it shows that the Mueller maneuver may be used as a clinical screening device for predicting the probability of having severe OSAS, and therefore be prioritized for a sleep study.

Other studies have shown correlations between upper airway and OSAS. Santiago-Recuerda et al. [20] conducted a study on 40 morbidly obese women to determine the relationship of upper airway changes and OSAS. They found that the oropharyngeal area at maximal inspiration was negatively correlated with AHI (*r* = −0.423, *p* = 0.044). This study used computed tomography assessments of the upper airway. More recently, Kum et al. [21] used the Mueller maneuver to measure collapse at the retrolingual level and found the LPW obstruction site to be correlated with AHI in OSAS patients. A similar obstruction grading scale was used in the present study; however, our results indicate an important relationship between the BOT and OSAS in addition to the relationship between the LPW and OSAS. To our knowledge, the present study is the first showing a relationship between BOT collapse, LPW collapse and OSAS severity using our graded obstruction scale based on the ease of the MM technique.

## Conclusions

In this study, the Mueller maneuver helped identify patients with severe sleep apnea based on UA collapse. MM cannot be used to diagnose OSAS, but can be a valuable tool to help the physician estimate the severity of sleep apnea and the urgency to obtain a sleep study. This examination can be performed in the physician’s office as a part of the routine head and neck examination. It allows for physicians to identify patients with significant collapse at the BOT and LPW, and as a result prioritize them for a formal sleep study.

## Abbreviations

OSAS: Obstructive sleep apnea syndrome
UA: Upper airway
VP: Velopharynx
BOT: Base of Tongue
LPW: Lateral pharyngeal walls
MM: Mueller maneuver
ESS: Epworth Sleepiness Scale
AASM: American Academy of Sleep Medicine
SD: Standard deviation
CI: Confidence interval
r: Correlation Statistic
OR: Odd ratio
MRI: Magnetic Resonance Imaging
CT: Computed Tomography
Pcrit: Critical pressure measurement
NEP: Negative expiratory pressure technique
DISE: Drug-induced sleep endoscopy
UPPP: Uvulopalatopharyngoplasty
